# Protective and therapeutic effects of *Trianthema portulacastrum* against atherosclerosis in male albino rats via G-protein-coupled receptor 124

**DOI:** 10.1186/s13568-019-0901-7

**Published:** 2019-10-31

**Authors:** Haoyu Wu, Tianjiao Gao, Yiwei Cao, Jiayu Diao, Fengjun Chang, Jie Qi, Congxia Wang

**Affiliations:** 1 Department of Cardiology, The Second Affiliated Hospital, Xi’an Jiaotong University, Xi’an, 710004 Shaanxi China; 2 Department of Cardiology, Shaanxi Provincial People’s Hospital, Xi’an, 710068 Shaanxi China; 3 Department of Gastroenterology, The Affiliated Children Hospital, Xi’an Jiaotong University, Xi’an, 710003 Shaanxi China; 4 Department of Functional Examination, Shaanxi Provincial People’s Hospital, Xi’an, 710068 Shaanxi China

**Keywords:** Diabetic rats, Atherosclerosis, Glucose, Cholesterol, Triglycerides

## Abstract

Atherosclerosis is a severe cardiovascular disease characterized by narrowing of the lumen, plaque formation, and blood flow turbulence as a result of cholesterol and lipid accumulation in the inner lining of arteries. Bishkhapra (*Trianthema portulacastrum* Linn.) is a well-known common weed belonging to the family *Aizoaceae*. Several bioactive compounds have been isolated from this weed and widely used against several diseases. The present study evaluated the protective and therapeutic efficacies of *T. portulacastrum* against atherosclerosis in a rat model. The animals were divided into the sham, control (diabetes- + atherosclerosis-inducing diet), 100 mg/kg *T. portulacastrum* treatment, 200 mg/kg *T. portulacastrum* treatment, and positive control groups. Blood glucose, cholesterol, triglyceride, and other lipid parameters, as well as the expression of G-protein-coupled receptor 124 (GPR124), were measured. Glucose, cholesterol, and triglycerides were significantly reduced to near normal levels. The serum levels of fibrinogen, sVCAM-1, and oxidized low density lipoproteins were substantially increased in control rats. Treatment with the *T. portulacastrum* extract reversed these levels to near normal levels. The mRNA expression of GPR124 was increased by 150% in the control group. However, treatment with *T. portulacastrum* extract decreased the mRNA expression up to 40% compared with the control group. Rats treated with 100 and 200 mg/kg *T. portulacastrum* extract showed a decrease in GPR124 protein expression by 9.5% and 33.3%, respectively. Taken together, the results suggest that an extract of *T. portulacastrum* is effective against atherosclerosis in streptozotocin-induced diabetic rats.

## Introduction

Atherosclerosis is a severe cardiovascular disease (Bader 2010) characterized by narrowing of the lumen, plaque formation, and blood flow turbulence resulting from cholesterol and lipid accumulation in the inner lining of arteries (Ye et al. 2013). Risk factors of atherosclerosis are abnormal lipid metabolism, dysfunction of arterial lining and inflammatory reactions (Jaipersad et al. 2014). An association between atherosclerosis risk and plasma lipid levels has been reported (Matsumoto et al. 2014). Zhang et al. (2013) reported that atherosclerotic plaque formation is caused by an inflammatory response to injury. Endothelial lining dysfunction in lesion-prone areas of the arterial vasculature may be an early indicator of atherosclerosis (Gimbrone and Garcia-Cardena 2016). It has also been reported that an increased blood level of low density lipoprotein (LDL) is the primary cause of atherosclerosis (Ference et al. 2017), and that the development of atherosclerosis, despite a low LDL level, is associated with several risk factors, including diabetes mellitus, smoking, genetic factors, and male sex (Aikawa et al. 2001). G-protein-coupled receptor 124 (GPR124) is an orphan receptor of the GPCR subfamily. Gong et al. (2018) reported that GPR124 increases the pathogenesis of atherosclerosis via activation of inflammation.

Plant-derived medicines have been used extensively for the treatment of several severe diseases (Sofowora et al. 2013). The World Health Organization reports that more than 80% of the population depends on plant-derived medicines for treatment (Ye et al. 2013). Bishkhapra (*Trianthema portulacastrum* Linn.) is a common weed belonging to the family *Aizoaceae*. Several bioactive compounds have been isolated from this weed and widely used against several diseases (Kumar et al. 2007). Protective effects of *T. portulacastrum* have been reported in rats fed an atherosclerosis-inducing diet (Shyam Sunder et al. 2010) and in rats induced to develop nephrotic syndrome (Karim 2012). Thus, the present study evaluated the protective and therapeutic efficacies of *T. portulacastrum* against atherosclerosis in rats.

## Materials and methods

### Rats

Male albino rats (180–210 g) were purchased from the animal house of Xi’an Jiaotong University, Xi’an, Shaanxi, and China. Animals were kept in standard rat polypropylene cages (435 × 290 × 150 mm; six rats per cage) and maintained under standard conditions of 12 h light/12 h dark at a relative humidity of 60 ± 5% and temperature of 25 ± 0.5 °C with food and water provided ad libitum.

### Preparation of plant materials

*Trianthema portulacastrum* plants were collected from a local region in Shanghai, China (Voucher specimen: 2018-341). Leaves were washed repeatedly in tap water, dried in sunlight, and ground into a fine powder. The prepared powder was packed into a Soxhlet apparatus and extracted with methanol (70%) in water for 22 h at 70 °C. The methanol extract was evaporated at 45 °C and then dried in a vacuum oven and stored for further use (Anil et al. 2018).

### Experimental diabetes

Experimental diabetes was induced by single intraperitoneal administration of streptozotocin (pH 4.5; 45 mg/kg in citrate buffer) to fasted rats (12 h). Streptozotocin-administered rats exhibited a hyperglycemia (glucose level: 250 mg/dL) within 48 h (Graham et al. 2011).

### Induction of atherosclerosis

Experimental atherosclerosis was induced by feeding rats an atherosclerosis-inducing diet. The diet contained 1.5 mL olive oil, 40 mg cholesterol, and 8 mg vitamin D2. The diet was given to rats daily for 5 days (Sharma et al. 2010).

### Experimental groups

The rats were divided into the following groups: sham, control (diabetes- + atherosclerosis-inducing diet), 100 mg/kg *T. portulacastrum* treatment, 200 mg/kg *T. portulacastrum* treatment, and positive control (600 μg/kg glibenclamide). Sham and control rats were given normal saline, whereas treated rats received the *T. portulacastrum* extract or glibenclamide (1 mL). The treatment was continued daily for 45 consecutive days.

### Biomarkers

The levels of blood glucose, total cholesterol, triglycerides, high density lipoprotein-cholesterol (HDL-C), LDL-cholesterol (LDL-C), and very low density lipoprotein-cholesterol were measured according to previously described methods (Wang et al. 2010; Aberare et al. 2011). Plasma fibrinogen and sVCAM-1 levels were measured using an enzyme-linked immunosorbent assay kit. Oxidized LDL and nitric oxide (NO) end products were determined according to a previously described method (Bryan and Grisham 2007; Itabe 2012). Apolipoprotein (Apo)-A and Apo-B levels were measured according to a previously described method (Cho et al. 2012).

### RT-PCR

Total RNA was isolated from heart tissue, and the RNA integrity was determined by gel electrophoresis. The RNA purity was determined by absorbance measurements at 260 nm. To produce cDNA, an oligo dT primer (0.5 ng), 10 mM dNTPs (2 µL), reverse transcriptase (100 U), and 5 × RT buffer (4 µL) were added to the total RNA (1 μg) in PCR tubes. The PCR tubes were incubated in a thermal cycler for 1 h at room temperature and then for 10 min at 90 °C. The relative mRNA expression of GPR124 was determined by RT-PCR (Table 1) according to Masatoshi et al. (2001).

**Table 1:** List of RT-PCR primers used in this study

S. No	Gene name	Forward primer	Reverse primer
1	GPR124	5′-AGCAAGAGGGGATTTCACAAT-3′	5′-GGTCGTTCTACTGGGCTGATT-3′
2	GAPDH	5′-GGTCACCAGGGCTGCTTTT-3′	5′-ATCTCGCTCCTGGAAGATGGT-3′

### Immunohistochemistry

Heart tissues were dissected and sectioned. The tissue sections were then fixed in formalin, embedded in paraffin, deparaffinized, and rehydrated with xylene using a graded alcohol series. A 3% hydrogen peroxide solution was added to inhibit endogenous peroxidase activity, followed by 2% bovine serum albumin to block nonspecific binding sites. Heart tissues were incubated with an anti-GPR124 antibody (1:300 dilutions, ab198817; Abcam, Cambridge, UK) overnight and then with a horseradish peroxidase-conjugated goat anti-rat antibody (1:300 dilutions, ab6721, Abcam) for 60 min (Balic et al. 2011). Immunofluorescence was visualized using a confocal microscope to assess GPR124 expression.

### Statistical analysis

Experimental results are expressed as the mean ± standard deviation. The results were compared by one-way analysis of variance, followed by Tukey’s post hoc test. The differences between the control and treated samples were considered significant at *P* < 0.05.

## Results

**Fig. 1:**
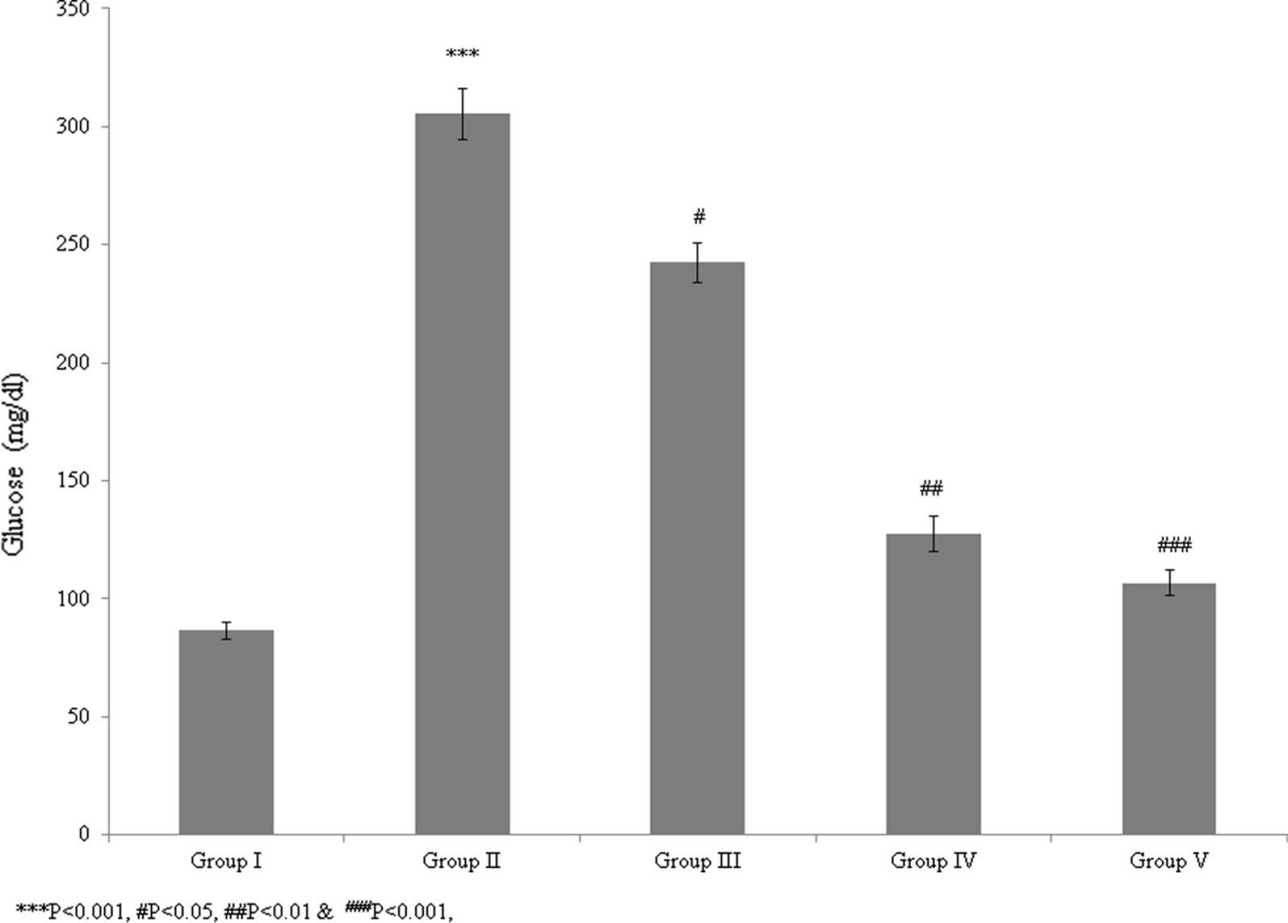
Protective effect of a *T. portulacastrum* extract on serum blood glucose levels (mg/dL) in normal diabetic rats. ***P < 0.001 vs. sham; ^#^*P* < 0.05, ^##^*P* < 0.01, and ^###^*P* < 0.001 vs. control. N = 6

**Fig. 2:**
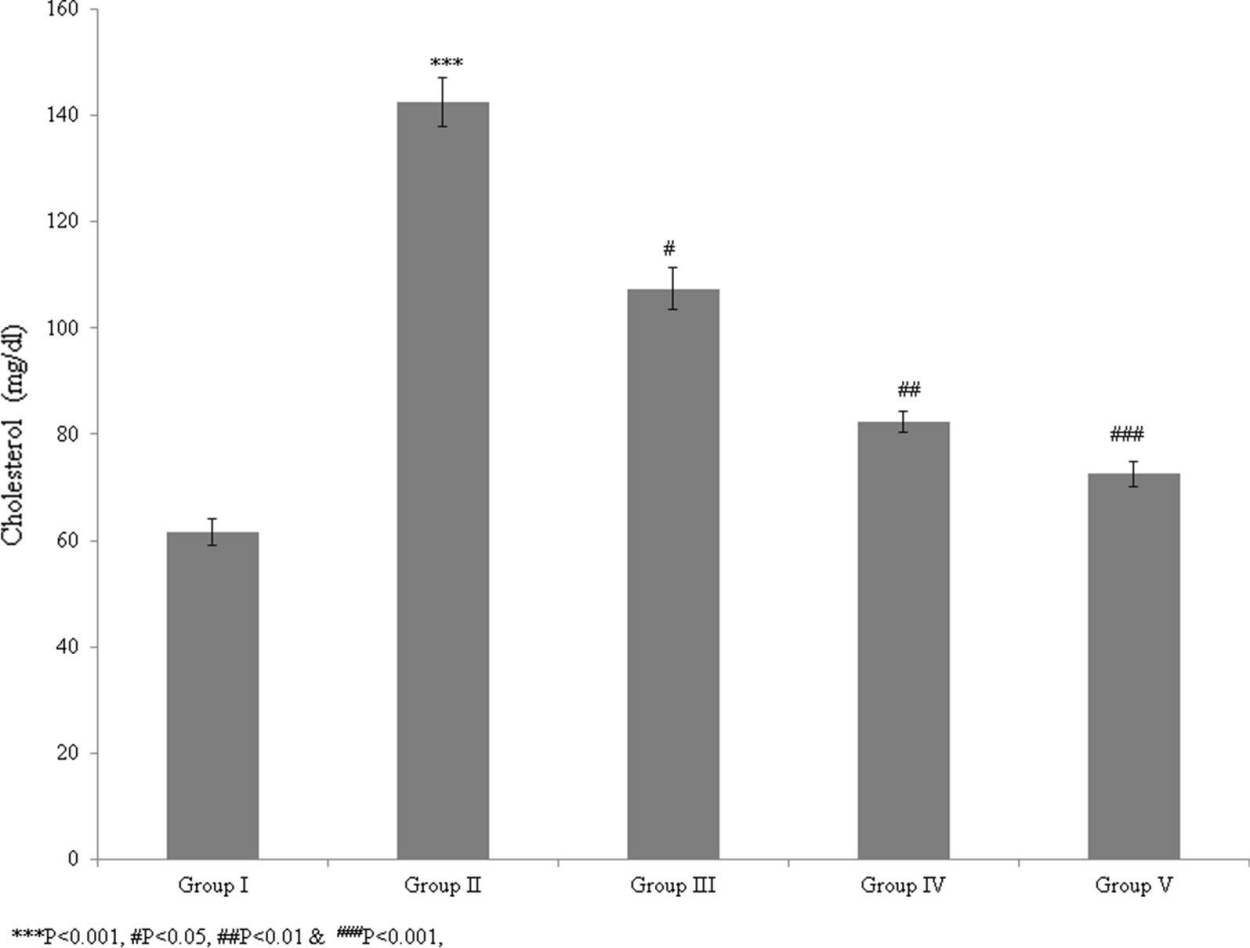
The protective effect of a *T. portulacastrum* extract on blood cholesterol levels (mg/dL) in normal diabetic rats. ***P < 0.001 vs. sham; ^#^*P* < 0.05; ^##^*P* < 0.01; ^###^*P* < 0.001 vs. control. N = 6

**Fig. 3:**
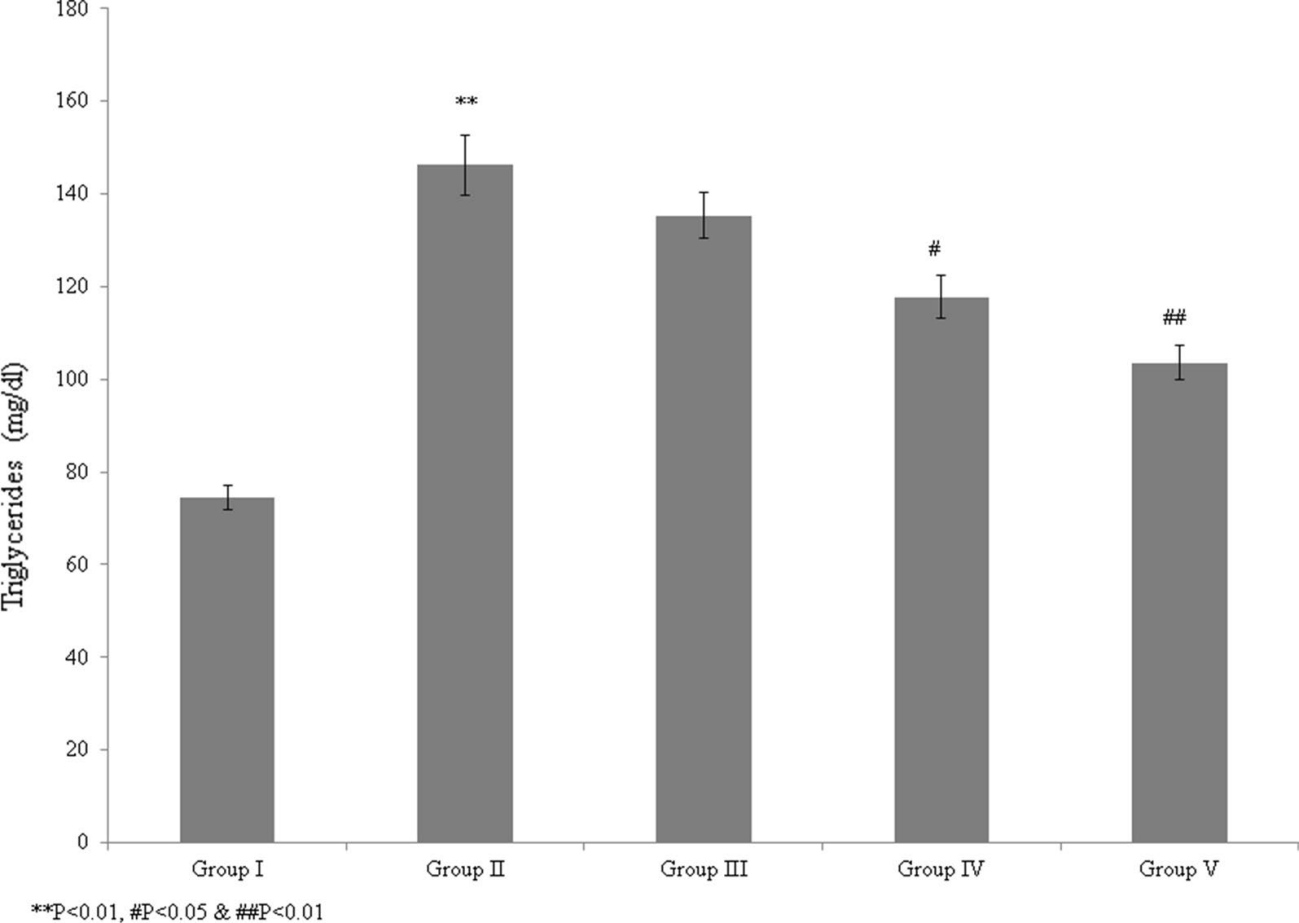
The protective effect of a *T. portulacastrum* extract on triglyceride levels (mg/dL) in normal diabetic rats. **P < 0.01 vs. sham; ^#^*P* < 0.05 and ^##^*P* < 0.01 vs. control. N = 6

In this study, we evaluated the protective and therapeutic efficacies of *T. portulacastrum* against atherosclerosis in a rat model. The blood glucose level was significantly reduced by 20.6% and 58.3% in control rats supplemented with 100 and 200 mg/kg *T. portulacastrum* extract, respectively (Fig. 1, *P* < 0.05), and by 65% in rats treated with glibenclamide, compared with untreated rats (Fig. 1; *P* < 0.05). The total cholesterol level was increased by 131.7% in the diabetic rats but was significantly reduced by 24.6% and 42.2% in the control rats supplemented with 100 and 200 mg/kg *T. portulacastrum* extract, respectively (Fig. 2; *P* < 0.05), and by 49% in rats treated with glibenclamide, compared with the untreated controls (Fig. 2, *P* < 0.05). The triglyceride level was increased by 96.2% in diabetic rats but was significantly reduced by 7.5% and 19.4% in control rats supplemented with 100 and 200 mg/kg *T. portulacastrum* extract, respectively (Fig. 3; *P* < 0.05), and by 29.2% in rats treated with glibenclamide, compared with the untreated animals (Fig. 3, *P* < 0.05).

The serum lipid profiles of the sham, control, extract, and glibenclamide groups are listed in Table 2. Diabetic rats showed increased LDL-C and decreased HDL-C levels compared with the sham rats (Table 2; *P* < 0.05). However, treatment with the *T. portulacastrum* extract significantly prevented these effects, with nearly normal LDL-C and HDL-C levels observed in treated rats (Table 2; *P* < 0.05). Serum levels of fibrinogen, sVCAM-1, and oxidized LDL were significantly increased in diabetic rats compared with the sham rats (Tables 3 and 4; *P* < 0.05) but were near normal levels in the rats treated with the *T. portulacastrum* extract (Tables 3 and 4; *P* < 0.05).

**Table 2:** Protective effect of *T. portulacastrum* extract on lipid profile in streptozotocin induced diabetic rats

Parameter	Group I	Group II	Group III	Group IV	Group V
HDL-C	34.8 ± 2.1	18.6 ± 1.2**	20.7 ± 1.4	28.4 ± 2.1^#^	30.2 ± 1.9^##^
LDL-C	15.6 ± 1.1	67.4 ± 3.7***	58.1 ± 3.5	44.5 ± 2.4^#^	30.3 ± 1.4^###^
VLDL-C	14.3 ± 0.7	28.5 ± 1.3**	25.3 ± 1.4	20.3 ± 1.2^#^	18.4 ± 1.1^##^

**P < 0.01, ***P < 0.001, ^#^*P *< 0.05, ^##^*P *< 0.01 and ^###^*P *< 0.001

**Table 3:** Protective effect of *T. portulacastrum* extract on endothelial dysfunction streptozotocin induced diabetic rats

Parameter	Group I	Group II	Group III	Group IV	Group V
Apo-A	–	41↓	8.2↑	18.3↑	23.2↑
Apo-B	–	23.5↑	7.3↓	16.3↓	18.3↓
NO levels (μmol/L)	–	28.3↓	13.3↑	18.2↑	26.5↑
Ox-LDL (μmol/L)	–	37.3↑	8.4↓	19.2↓	24.3↓

↑:  % increase, ↓:  % decrease

**Table 4:** Protective effect of *T. portulacastrum* extract on lipid profile in streptozotocin induced diabetic rats

Parameter	Group I	Group II	Group III	Group IV	Group V
sVCAM-1 (ng/mL)	542.3 ± 11.4	1356.4 ± 16.2***	1104.4 ± 13.2^#^	712.3 ± 10.5^##^	643.3 ± 11^###^
Fibrinogen (ng/mL)	2.05 ± 0.11	3.1 ± 0.15**	2.8 ± 0.14	2.5 ± 0.13^#^	2.4 ± 0.12^##^

**P < 0.01, ***P < 0.001, ^#^*P *< 0.05, ^##^*P *< 0.01 and ^###^*P *< 0.001

**Fig. 4:**
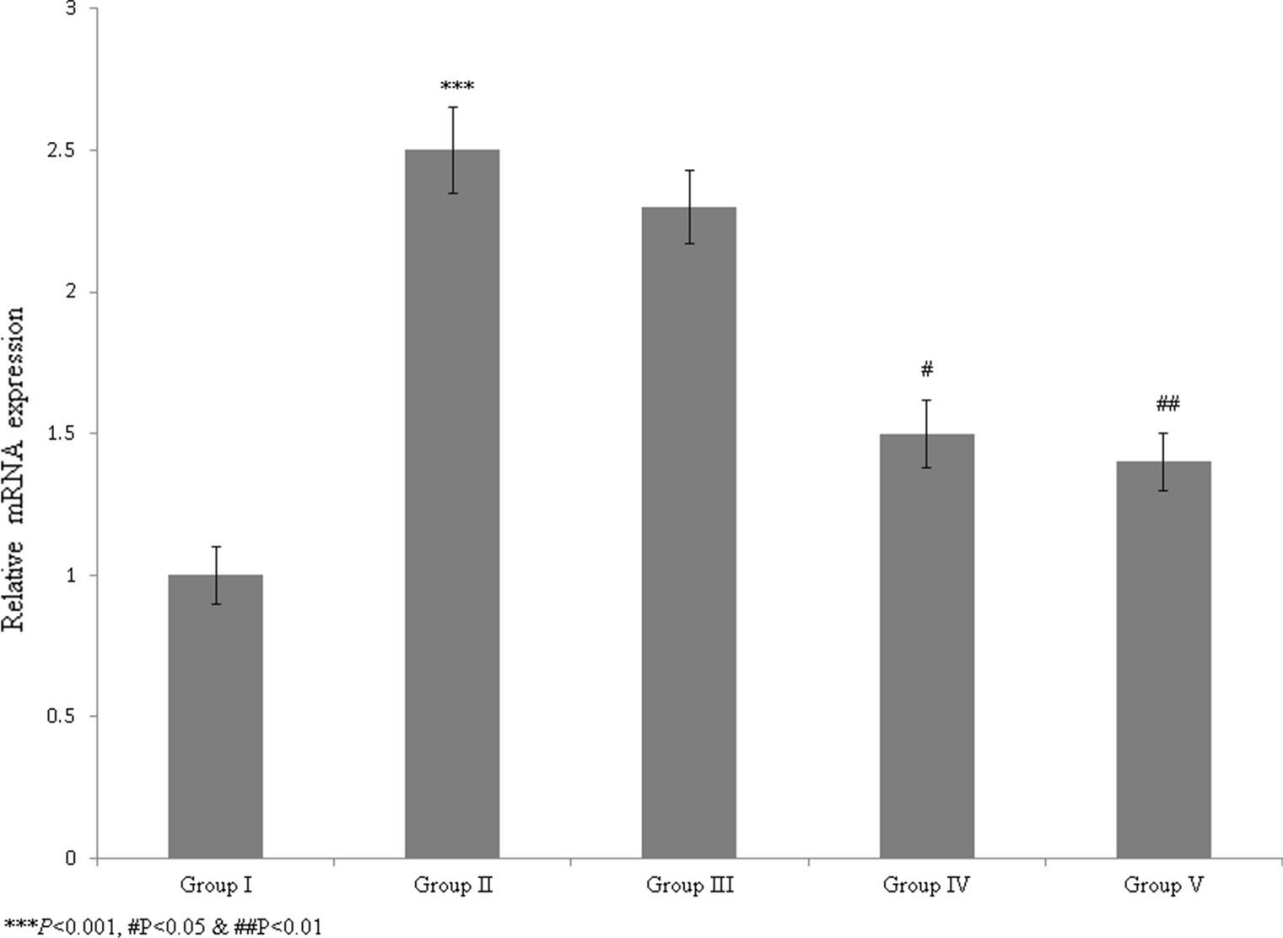
The protective effect of a *T. portulacastrum* extract on mRNA expression of GPR124 in normal diabetic rats. ***P < 0.001 vs. sham; ^#^*P* < 0.05 and ^##^*P* < 0.01 vs. control. N = 6

**Fig. 5:**
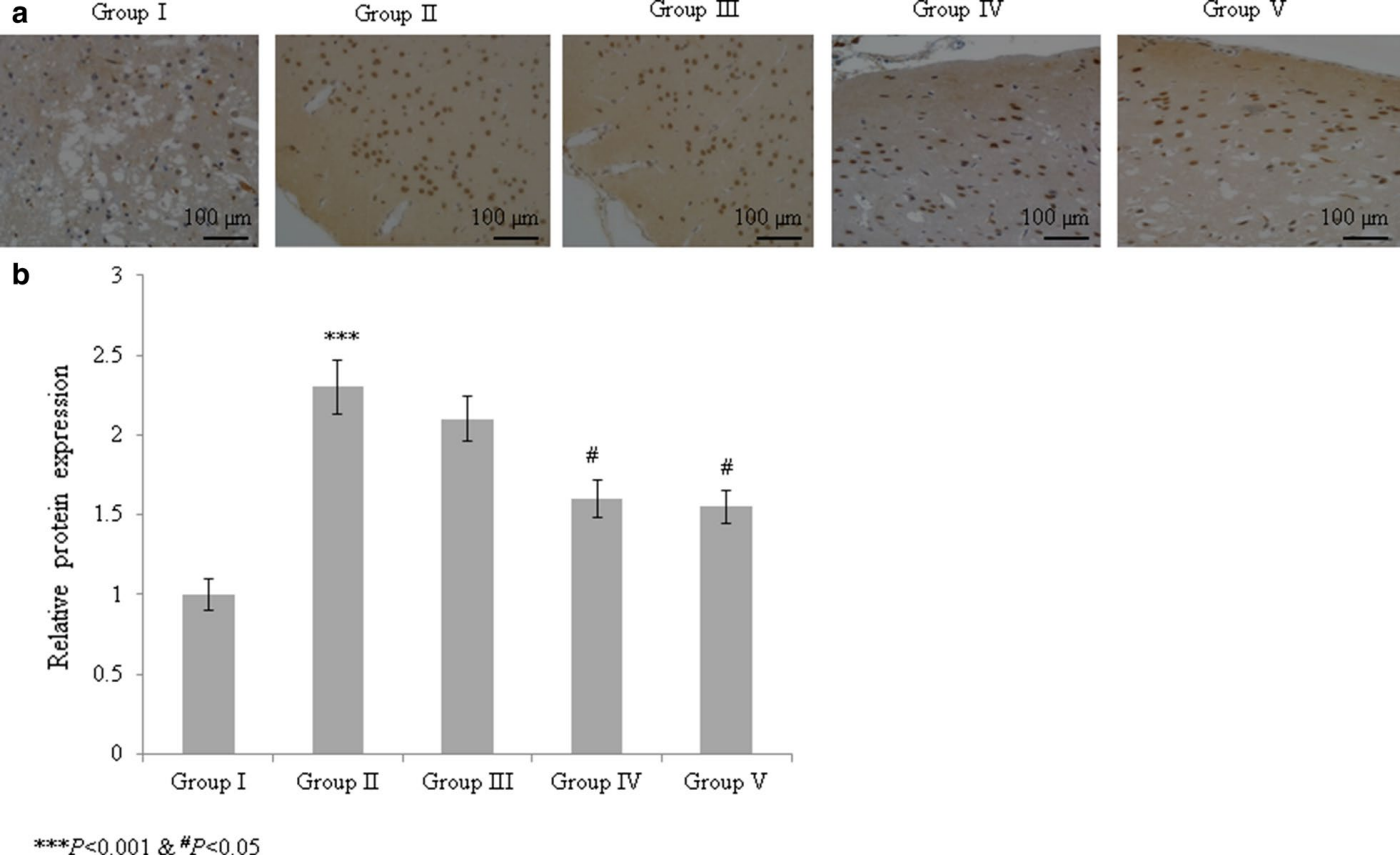
The protective effect of a *T. portulacastrum* extract on protein expression of GPR124 in normal diabetic rats. **a** Immunohistochemical expression of GPR124 and **b** protein expression of GPR124. ***P < 0.001 vs. sham; ^#^*P* < 0.05 vs. control. N = 6. Scale bar is 100 µm

The NO level was significantly reduced in diabetic rats, but treatment with the *T. portulacastrum* extract in these rats significantly increased the NO level to nearly normal levels (Table 3; *P* < 0.05). Furthermore, treatment of diabetic rats with the *T. portulacastrum* extract significantly improved the Apo-A and Apo-B levels (Table 4; *P* < 0.05). The mRNA expression of GPR124 was significantly increased by 150% in diabetic rats compared with the sham rats (Fig. 4; *P* < 0.05). However, the rats treated with 100 and 200 mg/kg *T. portulacastrum* extract exhibited significantly reduced GPR124 mRNA expression, by 8% and 40%, respectively (Fig. 4; *P* < 0.05). The protein expression of GPR124 was substantially increased by 130% in diabetic rats compared with sham rats (Fig. 5; *P* < 0.05). However, the diabetic rats treated with 100 and 200 mg/kg *T. portulacastrum* extract exhibited significantly reduced GPR124 protein expression, by 9.5% and 33.3%, respectively (Fig. 5; *P* < 0.05).

## Discussion

In this study, we evaluated the protective and therapeutic effects of *T. portulacastrum* against atherosclerosis in a rat model. Atherosclerosis is a severe cardiovascular disease (Bader 2010) characterized by narrowing of the lumen, plaque formation, and blood flow turbulence resulting from cholesterol and lipid accumulation in the inner lining of arteries (Ye et al. 2013). Arterial lining dysfunction, abnormal lipid metabolism, and inflammatory reactions are the main risk factors for atherosclerosis (Jaipersad et al. 2014). Investigators have reported the association of the risk of atherosclerosis with levels of plasma lipids (Matsumoto et al. 2014). Zhang et al. (2013) have reported that atherosclerotic plaque formation is due to an inflammatory response to injury. Investigators have reported that dysfunctions of the endothelial lining of lesion-prone parts of the arterial vasculature were considered as early indicators of atherosclerosis (Gimbrone and Garcia-Cardena 2016).

An increase in the blood level of LDL is reported to be the primary cause of atherosclerosis (Ference et al. 2017), and the development of atherosclerosis, despite a low level of LDL, is associated with several risk factors, including diabetes mellitus, smoking, genetic factors, and male sex (Aikawa et al. 2001). Several bioactive compounds have been isolated from the *T. portulacastrum* weed and widely used against several diseases (Kumar et al. 2007). Investigators have reported the protective effect of *T. portulacastrum* in rats fed an atherosclerosis-inducing diet (Shyam Sunder et al. 2010). Anreddy et al. (2010) reported the hypolipidemic and hypoglycemic activities of *T. portulacastrum* in alloxan-induced diabetic rats. Investigators have also reported the protective effect of *T. portulacastrum* in adriamycin-induced nephrotic syndrome (Karim 2012). Qadir et al. (2018) reported the potential role of medicinal plants as anti-atherosclerotic agents. Gong et al. (2018) reported that GPR124 increases the pathogenesis of atherosclerosis via activation of inflammation. In the present study, treatment of diabetic rats with a *T. portulacastrum* extract significantly reduced the expression of GPR124, which confirmed the protective role of this extract against atherosclerosis. Taken together, the results showed that an extract of *T. portulacastrum* was effective against atherosclerosis in streptozotocin-induced diabetic rats.

## Data Availability

Corresponding author could provide the all experimental data on valid request.
